# L2 acquisition of *Wh-*interrogatives at the syntax-discourse interface: interface hypothesis again

**DOI:** 10.12688/f1000research.133802.1

**Published:** 2023-09-25

**Authors:** Mohammed Shormani

**Affiliations:** 1 Department of English Studies, University of Cyprus, Nicosia, Cyprus; 2 English Studies, Ibb University, Ibb, 70270, Yemen

**Keywords:** L2 acquisition, wh-interrogatives, Interface Hypothesis, Syntax-discourse Interface, D-linking, (pronoun) resumption

## Abstract

This study sets out to answer one major question: do linguistic phenomena relating to syntax-discourse interface constitute difficulty for Yemeni learners of English? It presents data from an experiment on the acquisition of L2 English *wh-*interrogatives by L1 Yemeni Arabic speakers, aiming to provide empirical evidence either in support of the Interface Hypothesis (IH) or against it. Two learner groups, intermediate and advanced, were recruited as participants of the study, and a native speaker group of (British) English was also recruited as the control group. The advanced group learners have a near-native proficiency in English. The data utilized consisted of 20 (D-)iscourse linked and non-d-linked *wh-*interrogatives presented to the three groups in the form of a (decontextualized) bi-modal multiple-choice paced judgement task. Results showed that both learner groups, specifically the advanced learners, performed near-native like in the non-d-linked, but far short of near/native-like performance in the d-linked *wh-*interrogatives. The study concluded that L2 learners’ English is still vulnerable at the syntax-discourse interface, hence supporting the IH.

## 1. Introduction

Recent second language acquisition (SLA) research has witnessed a remarkable shift in scope and orientation, focusing more on the acquisition of interfaces of syntax with other modules of the grammar rather than on the syntax proper. Studies conducted in this line of thought focused on examining second language (L2) acquisition of two or more typologically different languages. The interest in examining two or more typologically different languages in such bilingual SLA studies is to see to what extent these (typological) differences cause difficulty for L2 learners. The differences may include a “partial structural overlap across the two languages where language A uses construction X in context X and construction Y in context Y, while language B uses construction X in both context X and context Y” ( Sorace and Serratrice 2009: 196). In other words, such studies aim to identify the influence of this bilingual overlap and whether, and to what extent it causes non-convergence from native or near-native L2 endstate grammar. However, the focus of these studies may be different in the sense that each study may focus on one particular aspect to investigate. For instance, some studies focus on the ability of L2 learners to re-set the parameters according to L2 they are acquiring (see *e.g.* Belletti *et al.* 2007). Some others investigate L2 learners’ residual optionality in interpreting uninterpretable features in their endstate L2 grammars (see *e.g.* Tsimpli and Dimitrakopoulou 2007). And some others aim to find out where these difficulties lie and which module of the grammar they belong to (see *.e.g.,* Sorace 2005, 2006; Sorace and Serratrice 2009): do they belong to syntax proper, or syntax interfaces with other cognitive domains?

This study examines the availability of the Interface Hypothesis (IH), first proposed by Sorace and colleagues ( Sorace and Filiaci 2006) that L2 learners’ endstate grammars are vulnerable at the syntax-discourse interface by first language (L1) Yemeni Arabic (YA) learners, who acquire English as L2, through examining the participants’ rejection of resumptive pronouns in English ungrammatical *wh-*interrogatives and acceptability of gaps/empty categories in grammatical ones. The study concerns primarily two sets of *wh-*interrogatives: grammatical and ungrammatical, at two main levels, namely (D-)iscourse linking and non-d-linking conditions, and in terms of subject and object contexts. It involves three groups of participants ( *n*=41): intermediate learner group (IMG, *n*=17), advanced learner group (AVG, *n*=14) and control group (NSG, *n*=10). The intermediate learners are BA English students, at their fourth year, and the advanced learners are MA English students (after completing three semesters of courses, and before commencing writing their MA Theses). Both learner groups are native speakers of YA, and the criterion followed to determine their proficiency in English is the Online University of Michigan grammar (placement) Test (OUMG) parts 1-4. The control group are native speakers of (British) English, MA students at the University of Essex.

The reason why I study this phenomenon is threefold: first, YA is a null/ *pro* subject language, while English is not. ^1^ Therefore, *wh-*question formation mechanism is expected to be different in both languages. For example, while YA uses base-generation strategy, English uses *wh-*movement strategy. ^2^ These two different mechanisms lead to two different resumption options: YA accepts resumptive pronouns in *wh-*interrogatives, English does not. Second, since YA typologically differs from English, YA (and its speakers) provides a good model for an experimental study. Thus, it is expected that L1 ( *i.e.*YA) interference may have a role to play in the difficulty encountered by YA learners of L2 English. And third, the paucity of studies in Yemeni context that tackle this important aspect of L2 acquisition research. To the best my knowledge, there has been no study in the literature investigating L2 acquisition at the syntax-discourse interface by L1 YA learners of English as L2. Thus, this study serves to fill this gap, by giving empirical support to the IH in SLA and contributing to the ongoing controversy on this unprecedented hypothesis.

The study not only provides strong support to the IH’s findings, but also for its further refinements that, while it is relatively easy to acquire linguistic phenomena related to the core syntax and those related to syntax and internal interfaces such as syntax-semantics, acquiring linguistic phenomena related to syntax and external interfaces like syntax-discourse is vulnerable even at advanced stages of L2 acquisition. The results show that whereas both groups, specifically advanced learners, performed near-native like at the syntax proper (non-d-linking condition), they were far short of native or near-native competence at the syntax-discourse interface (d-linking condition). Their performance was also better in object condition than in subject condition.

The article is set up as follows. Section 2 lays out the theoretical foundations of SLA at the interfaces, reviewing some important related studies, specifically those supporting the IH. Section 3 articulates the typological syntactic differences between *wh-*interrogative formation in YA and English, placing emphasis specifically on the *wh*-mechanism and resumption strategy used in both languages. Section 4 spells out the design and methods of the study, focusing among other things on the participants of the study, the nature of the data utilized, instrument used and analysis of the data collected from the questionnaires administered to the three groups. Section 5 presents the results, tabulating these results in terms of d-linked and non-d-linked, and subject and object *wh*-interrogatives, and presenting the One-way ANOVA and Post hoc Scheffe analyses of the findings at the level ( *p* = .05). Section 6 discusses the results reached in terms of three aspects, namely learners’ performance in d-linking *versus* non-d-linking conditions, their performance in object *versus* subject conditions, and Access to UG and L1 interference, and Section 7 concludes the article.

## 2. L2 acquisition at the interfaces

Within earlier generative approaches to syntax, Principles and Parameters (P&P) framework, ^3^ for example, SLA studies focused on the role of Universal Grammar (UG), and whether L2 leaners still have access to it in the acquisition of L2 (or even Ln). There are actually three positions: i) Full Access (see *e.g.* White 1986, Cook 1988; Flynn 1996; Shormani and AlSohbani 2015), ii) Partial Access (see *e.g.* Hawkins and Chan 1997; Smith and Tsimpli, 1995), and iii) No Access (see *e.g.* Bley-Vroman 1989). ^4^ While it was somehow difficult in P&P Framework to account for the difficulties encountered by (adult) L2 learners, and more importantly where (exactly) they lie within L2 grammars ( *i.e.* either within the core syntax or within its interfaces with other high cognitive domains that are not part of the core grammar), recent conceptions and developments in generative approaches to syntax such as the Minimalist Program ( Chomsky 2000, 2008), cartography approaches (see *e.g.* Cinque 2002; Rizzi 2004; Belletti *et al.* 2007), ^5^ discourse interface studies (see *e.g.* Reinhart 2006; Erteschik-Shir 2007; Frascarelli 2007; Shormani 2017) explicitly assist SLA scholars to account for the non-ultimate (or ultimate) attainment, the failure of native-like competence.

From a minimalist perspective, consider feature (un)interpretability; for instance, it is held that interpretable features at the syntax–discourse interface involve considerable difficulty for L2 learners ( Sorace 2005). For others, (see *e.g.* Tsimpli and Dimitrakopoulou 2007), however, it is uninterpretable features that constitute considerable difficulty for L2 leaners at the syntax-discourse interface, which results in variability in L2 endstate grammar. It was reported that these higher interface domains can also be extended to interpret “delays” experienced in L1 acquisition by children ( *e.g.* Platzack 2001) and L1 attrition ( *e.g.* Chamorro and Sorace 2018). Given this, it was not surprising to find evidence of variability or residual optionality in adult L2 leaners’ endstate grammar (see *e.g.* Sorace 2000, 2005, 2006, 2011, 2016; Tsimpli and Dimitrakopoulou 2007; Tsimpli and Mastropavlou 2007; Sorace and Serratrice 2009, Sorace et al., 2009).

Cartography approaches and discourse studies also help scholars identify where the difficulty encountered by L2 learners lies, in that they make explicit the structural representations beyond the syntax proper, or relating it to other cognitive domains. For example, cartography approaches make available linking the syntax proper with discourse/pragmatics interface (see *e.g.* Shormani 2017). Discourse has been identified in the left-periphery of constituent structure, specifically with the informational/coda structure, while the syntax proper with the propositional structure, the C-domain and T-domain, respectively (see *e.g.* Rizzi 1997, 2004, 2006; Cinque 2006; Shormani 2017; Shormani and Qarabesh 2018). To represent the discourse, the CP layer has been proposed to project to three (and sometimes four including FiniteP) projections, namely ForceP, TopicP, and FocusP ( Rizzi 1997, 2004, 2006; Cinque 2006), and sometimes even SpeakerP/AddresseeP (see *e.g.* Espinal 2013). In Rizzi’s (1997: 283) own words, the C-domain is “the interface between a propositional content (expressed by IP) and the superordinate structure (a higher clause or, possibly, the articulation of discourse, if we consider a root clause).” Thus, TopicP, for instance, has been referred to as a projection structurally representing left-dislocation phenomena such as clitic-left dislocation, or topicalization, which is a discourse property. Several studies have tackled this aspect to account for the difficulty encountered by L2 learners at the syntax-discourse interface (see *e.g.* Tsimpli and Sorace 2006; Dugarova 2014; Chamorro and Sorace 2018; Smeets 2018).

Thus, such recent conceptions and developments give SLA scholars enough room to interpret the difficulties encountered by L2 learners, as well as L1 acquirers alike, like the residual optionality, deficit parsing or misanalyzing of L2 structures, variability, permanent fossilization, etc. in adult L2 acquisition (cf. also Long 2003; Shormani 2013). For example, Sorace and colleagues ( Sorace and Filiaci 2006) propose the Interface Hypothesis in an attempt “to explain the non-convergence and optionality revealed in very advanced adult second language learners in the comprehension and production of certain structures” ( Chamorro and Sorace 2018: 2). The interface Hypothesis states that learners’ endstate knowledge of L2 is vulnerable and this vulnerability is due to interface effects ( Sorace and Filiaci 2006; Sorace and Serratrice 2009). That L2 advanced learners encounter considerable difficulty acquiring linguistic phenomena at the syntax-discourse interface, according to Sorace and colleagues, is due to the fact that this interface is a higher level of cognitive domains which requires high processing to be acquired.

The term *Interface* has been used to signify a level at which a module of the grammar is linked with other cognitive domains such as syntax-semantics, syntax-morphology, syntax-discourse (see *e.g.* Sorace 2005, 2006, 2011, 2016; Sorace and Serratrice, 2009). There are two types of interface: internal and external. The former refers to “those between narrow syntax and the other linguistic modules (phonology, morphology, semantics) and external interfaces, those between syntax and other cognitive modules” ( Slabakova and Ivanov 2011: 638). Put differently, internal interfaces are said to be between internal modules of the grammar such as syntax-semantics, and syntax-morphology, while external interfaces take place between syntax and external cognitive domains such as syntax-discourse, syntax-pragmatics.

As for the difference between internal and external interface, Tsimpli and Sorace (2006: 653) postulate that “[t]he distinction between the two interfaces is based on the assumption that the syntax-discourse interface is a ‘higher’ *level of language use*, integrating properties of language and pragmatic processing, whereas syntax-semantics involve formal properties of *the language system alone*” (emphasis mine). Furthermore, Sorace and Serratrice (2009) argue that the difference between internal and external interfaces lies in that the former involves operations internal to the ‘core computational system’, while the latter involves operations external to this computational system. ^6^ In their own words, “the syntax-semantic interface involves formal features and operations within syntax and Logical Form, whereas the syntax-discourse interface involves pragmatic conditions that determine appropriateness in context” (p. 197). The overall view held regarding these two types of interfaces, and which causes difficulty for L2 learners is that external interfaces are more difficult to acquire than internal ones. Sorace (2011: 1) writes: “language structures involving an interface between syntax and other cognitive domains are less likely to be acquired completely than structures that do not involve this interface” (see also Chamorro and Sorace 2018). ^7^ The claim is that external interfaces require high processing because they lie out of the core computational system, as has been alluded to above. In what follows, I will discuss some example studies whose results provide support to the IH.

The IH effects, as well as the difference between internal and external interfaces, were supported by empirical studies cross-linguistically with a wide range of L1s and L2s (see *e.g.* Sorace 2006, *et seq*; Tsimpli and Sorace 2006; Sorace and Serratrice 2009; Belletti *et al.* 2007; Antonova-Ünlü 2015; Chamorro and Sorace 2018; Smeets 2018). For example, Tsimpli and Sorace (2006) investigate the interface hypothesis in a study involving 27 adult Russian learners of Greek. They investigated learners’ ability to distinguish between focus (a syntax-semantics interface property) and clitic-left dislocation, or topicalization (a left-periphery, or more specifically a syntax-discourse interface property). The results indicate that the leaners involved were able to analyze focus data, but not left-dislocated clitics. They conclude that while the syntax-semantics interface is relatively (completely) acquirable, the syntax-discourse interface is vulnerable.

Furthermore, Sorace and Filiaci (2006) conducted a study examining the Anaphora resolution in near-native speakers of Italian. They recruited a control group of monolingual Italian native speakers and a group of native English speakers who had attained near-native proficiency in Italian. Their study supported the IH, pointing out that the residual optionality they found is still persistent at the syntax–discourse interface, which may be attributed to “indeterminacy”, failing to link pronouns to their antecedents. Similar findings were also reported by Belletti *et al.* (2007) who conducted a study to examine the null subject parameter (which is a discourse related phenomenon) *pro versus* overt subject pronoun in L2 Italian by 17 learners whose native language is (American) English. They concluded that there is significant divergence between L2 learners and native speakers, attributing this divergence to two sources: i) the visible outcome is the overproduction of overt subject pronouns, and the misinterpretation of overt subject, and ii) the unsystematic use of the low focus position in the VP-periphery of the clause dedicated to the new-information postverbal subjects ( Belletti *et al.* 2007: 682). Further, Antonova-Ünlü (2015) conducted a study whose focus was L2 acquisition of Case markers in Turkish by highly proficient learners of L1 Russian. According to Antonova-Ünlü, Case markers in Turkish involve syntax-semantics/morphology interface and syntax-discourse interface, the latter of which requires pragmatic understanding for accurate interpretation. She concluded that while L2 learners easily acquire syntax-semantics/morphology interface materials, syntax-discourse interface linguistic phenomena, however, were vulnerable.

In addition, Tsimpli and Dimitrakopoulou (2007) conducted a study on the acquisition of English *wh-*interrogatives by L1 Greek learners. In this study, the authors examined the residual optionality in L2 acquisition, proposing the Interpretability Hypothesis, which states that uninterpretable features, but not interpretable features, constitute considerable difficulty for L2 learners. The study recruited two groups of learners, 21 intermediate and 27 advanced, and 26 native speakers of English, as a control group. The data utilized consisted of 30 *wh-*interrogatives (and 21 distractors). They examined resumption, d-linking and animacy effects on the responses of their participants. They found that while non-d-linked *wh-*questions were relatively easy to learn by both learner groups, d-linked ones were difficult. They concluded that L2 acquisition is vulnerable at the syntax-discourse interface, thus adding strong support to the IH. In addition, Smeets (2018) conducted a study examining near-native grammars at the syntax–discourse interface of two different domains of object movement in Dutch exhibiting two properties lied within syntax-semantics and syntax-discourse interfaces. In this study, the author recruited 56 participants, 15 of which were native speakers of Dutch, 16 native speakers of German and 25 native speakers of English. Her instrument was felicity judgment tasks and a truth value judgment task. The results showed that while syntax-semantics interface properties were easy to acquire, syntax-discourse were not so. However, she opines that for L2 convergence, discourse cues should be sufficiently salient in the input. She added that due to L1-L2 typological differences, “L2 learners may not fully acquire L2 discourse conditions on syntax when their L1 allows the relevant syntactic construction as well but does so in different discourse settings” (p. 21).

## 3. *Wh-*interrogatives in English and Arabic

Recall that YA is a null subject, or *pro*-drop language, while English is not. Therefore, it is expected that both languages differ in the way each forms *wh-*interrogatives. I will discuss the differences between English and YA in terms of d-linked [+DL] and non-d-linked [-DL] *wh-*interrogatives. ^8^ Section **3.1** addresses the former, and Section **3.2** tackles the latter.

### 3.1. D-linked wh-interrogatives

Consider (1a) which exhibits subject d-linked *wh-*interrogatives, and (1b) which exemplifies object d-linked ones.

(1) a. ʔayyan rajjaal qaal ʕali *(ʔinn-uh) jaaʔ ʔams?

     which man said Ali  that-he     came yesterday

     ‘Which man did Ali say (*that he) came yesterday?’

  b. ʔayyan dars qaal ʕali *(ʔinn-uh) katab*(-uh)?

     which lesson said Ali     that-he wrote-it

     ‘Which lesson did Ali say that he wrote (*it)?

As can be observed in (1), it is clear that in YA *wh-*questions the complementizer and the resumptive (clitic) pronoun attached to it *ʔinn-uh* is obligatory in both subject and object d-linked *wh-*questions, as in (1a) and (1b), respectively. As for subject d-linked *wh-*questions, English behaves exactly in the reverse manner in that the complementizer and the resumptive pronoun ‘that-he/it’ must be absent in English subject d-linked *wh-*interrogatives, as the English translation shows. This could be noted as the first difference between both languages. And to account for this difference, it is plausible to look at the mechanism each language uses in forming *wh-*questions. It is well-known that English uses movement strategy by moving the *wh-*word/phrase to Spec, CP, leaving a gap in the base (cf. *e.g.* Ross 1986; Chomsky 1977, 1995). However, YA uses base-generation strategy, *i.e.* the *wh-*word/phrase is base-generated in Spec, CP (see *e.g.* Demirdache 1991: 43; Boeckx 2003; Shormani 2015), with a matching resumptive pronoun in the base.

Regarding the object *wh-*interrogatives, YA uses the complex ‘that-he/it’ and a resumptive object pronoun. However, English does allow ‘that-he/it’, but does not allow object resumptive pronouns. This could be termed as the second difference between English and YA.

### 3.2. Non-d-linked wh-interrogatives

In this section, I address the differences between English and YA in the non-d-linked *wh-*interrogatives both in subject and object contexts exemplified in (2a) and (2b), respectively:

(2) a. manu qaal ʕali *(ʔinn-uh) jaaʔ ?

    who said Ali  that-he  came

    ‘Who did Ali say (*that he) came?’

  b. mu qaal ʕali *(ʔinn-uh) ʔaštri*(-uh) ?

    what said Ali  that-he  bought-it

    ‘What did Ali say (that) he bought (*it)?

In subject non-d-linked *wh-*questions, and unlike English, YA accepts the complex ‘that-he/it. ^9^ English, however, does not allow the complex ‘that-he/it’ in subject non-d-linked *wh-*questions. In object *wh-*questions, however, English differs from YA in that it does not allow the resumptive pronoun *it.*

Theoretically, the English properties found above are due to the fact that English does not allow *wh-*extraction out of a subject position, but it allows such extraction out of an object position, a phenomenon known classically as *that-t* effects (see *e.g.* Pesetsky 2017, for a recent discussion). For example, in the English translation in (2a), English does not allow either the complementizer *that*, the pronoun *he* or both *that-he* in subject *wh-*interrogatives. However, English allows the complementizer *that* in object position, but not the resumptive pronoun *it*, as the English translation of (2b) clearly indicates.

To conclude, the differences observed between English and YA in the way each forms *wh-*interrogatives could be summarized as follows:

In *wh-*interrogative formation,

1. YA uses the base-generation strategy while English uses the movement strategy,
2. YA accepts resumptive pronouns in both subject and object d-linked and non-d-linked *wh-*interrogatives, while English does not allow resumptive pronouns, English accepts gaps/empty categories, instead, and,
3. English exhibits *that-t* effects, while YA does not.

Based on these differences between YA and English, the hypotheses of the study are as follows:

a) Given (1) above, it is predicted that L1 YA learners of L2 English will not recognize the effects caused by the movement strategy in English, which will result in non-target performance,
b) Given (2), it is predicted that L1 YA learners of L2 English will accept resumptive pronouns in both d-linked and non-d-linked *wh-*interrogative, and,
c) Given (3), it is predicted that L1 YA learners of L2 English will be unsensitive to *that-t* effects, and hence inappropriately respond to *that-t* stances as well-formed structures.

## 4. The present study

### 4.1. Methods

**4.1.1. Ethical statement**

First, the ethical approval for this study was obtained from the Department of English Studies, Faculty of Arts, Ibb University, Yemen. Second, written Informed Consent Forms were presented to all the participants in the study, both the experimental groups, *viz.,* intermediate and advanced learners, and the control group. The Informed Consent Forms were presented along with the questionnaire, through which the consent was obtained from all the participants prior to their participation in the study. In the consent forms, it was clearly stated that their participation is *voluntary* and that all their responses and anonymous data will be used *only* for scientific research purposes. The participants were also assured that their participation will be acknowledged in the study.

**4.1.2. Participants**

The study involves three groups of participants ( *n*=41): control group/native speaker group (NSG), and two learner groups, intermediate group (IMG) and advanced group (AVG). The NSG involves 10 participants ( *n*=10), native speakers of (British) English, Masters of Arts (MA) students, studying at the University of Essex. The IMG consists of 17 participants ( *n*=17), BA students at the final (fourth) year. AVG consists of 14 ( *n*=14), MA students who completed their three semesters of courses (and before commencing writing their MA Theses). Both learner groups were recruited from Ibb University, Yemen. The Mean Age (M) of the three groups is as follows: NSG is 29.3 years, IMG 25.8 years and AVG 34.2 years. The IMG participants have studied English for 10 years. They studied English as a compulsory subject in schools for six years. They have also studied English for four years at the university in BA Degree. Furthermore, the AVG participants have studied English for about 12 years, as a compulsory subject in schools for six years, four years in BA Degree, and one and half years in MA program.

**4.1.3. Instrument**

The *wh-*interrogative data were presented to the participants in three forms: i) an online questionnaire constructed on Google.Forms for native speakers of English). The link of this instrument was sent to an MA student at the University of Essex, requesting her to ask 10 MA students to respond to the items of the questionnaire, ii) a hardcopy of the same questionnaire was administered to the IMG participants, and iii) a hardcopy of the same questionnaire was also administered to the AVG participants (see Shormani 2023).

The task was a (decontextualized) bi-modal paced judgement task, whereby the participants were asked to judge the acceptability/unacceptability of 20 *wh-*interrogatives on a 1-4 Linkert scale, ranging from *Natural* to *Very Odd.* The responses are *Natural, Ok, Odd* and *Very Odd.* Both intermediate and advanced learners were allotted 20 minutes to judge the 20 *wh-*questions, *i.e.* one minute for each *wh-*question. I tested these 20 *wh-*interrogatives considering d-linking *versus* non-d-linking, and subject *versus* object as conditions. *Groups* is the dependent variable. Concerning the acceptability of the *wh-*interrogatives, I count the options *Natural* and *Ok* as target, and *Odd* and *Very Odd* as non-target.

**4.1.4. Data utilized**

The data utilized in the study are *wh-*interrogatives, involving 20 *wh-*interrogatives. They involve 10 *wh-*interrogatives for d-linking and 10 for non-d-linking. Both categories involve both subject and object *wh-*interrogatives. Table 1 presents these categories along with their frequency.

**Table 1.** D-linked and non-d-linked *wh-*questions together with their frequency of occurrence.

Category	Subject *wh-*interrogatives	Object *wh-* interrogatives
D-linked	5	5
Non-d-linked	5	5

The data also involved 10 grammatical and 10 ungrammatical *wh-*interrogatives. Table 2 presents two examples for (+DL) *wh-*phrase, i.e. *Which-NP,* and two examples for each (-DL) *wh-*words, namely *Who* and *What.* (For the full set of *wh-*interrogatives along with number of occurrences, see Shormani 2023). ^10^

**Table 2.** Examples of the d-linked and non-d-linked *wh-*interrogatives.

D-linked data	Non-d-linked data
6 = *Which book do you think that Alia read it? 7 = Which team do you say will win the match?	1=Who did you say ran away? 2=*Who do you think that he saw Ali? 16=What did Ali suggest should be made clear to all students? 18= *What did you say that Alia wrote it when she saw her friend?

As Table 2 shows, three *wh-*words/phrases were utilized, namely *Who, What* and *Which-NP*; *Who* and *What* are non-d-linked *wh-*words, and *Which-NP* is a d-linked *wh-*phrase. *Who* and *What* represent subject and object non-d-linked *wh*-words, respectively, and W *hich-NP* represents subject and object d-linking.

The data utilized involved complex *wh-*interrogatives, complex in the sense that each consists of matrix and complement (embedded) clauses, and the participants were told about this fact. In the matrix clauses, verbs that require sentential complements were used. These are *say, think, suggest, remember*, etc. The responses were collected from the three questionnaires. The data were analyzed by the researcher using SPSS V. 27. One-way ANOVA was used to analyze the statistical differences between groups. As for comparisons within groups, a Post hoc Scheffe was used.

**4.1.5. Criterion**

The criterion that was utilized to determine the division of both experimental groups is the Online University of Michigan Grammar (placement) Test (OUMG) parts 1-4. The OUMG Test assesses different and several aspects of English grammar. ^11^ The participants were allotted 20 minutes to answer 40 multiple-choice questions, i.e. each question was given ½ minute. IMG’s minimum score was 67 % and maximum 78% accuracy, and AVG’s minimum score was 83 %, and maximum 91% accuracy (cf. Table 3). Based on these results, IMG participants were considered to have an intermediate level in English, and those in AVG an advanced level. Table 3 presents the results of the OUMG test.

**Table 3.** The results of OUMG test.

Group	*n*	Accuracy	
		minimum	maximum
NSG	10	-------	-----------
IMG	17	67%	78%
AVG	14	83%	91%

The variables tested in this study are presented in Table 4.

**Table 4.** Variables tested in the paced judgment task.

	D-linking	Resumption
Subject *wh-*interrogatives	*wh-*NP *wh-*word	+ resumptive - resumptive
Object *wh-*interrogatives	*wh-*NP *wh-*word	+ resumptive - resumptive

## 5. Results

The results collected from the acceptability judgment from the three groups are presented in Tables 5 and 6. The results are presented in two formats according to the conditions, *i.e.* d-linking and non-d-linking, and subject and object. I first count the participants’ responses for the d-linked *wh-*interrogatives in both subject and object contexts. Then I count the responses for the non-d-linked *wh-*interrogatives also in both subject and object contexts. For differences between groups, I used One-way ANOVA analysis for the differences between groups, and Post hoc Scheffe test was used for differences within groups.

**Table 5.** Performance in d-linked subject–object *wh-*interrogatives (percentages, with *n* in parentheses).

Group	Subject		Object	
	Target	Non-target	Target	Non-target
IMG	42.4% (36/85)	57.6% (49/85)	54.1% (46/85)	45.9% (39/85)
AVG	58.6% (41/70)	41.4% (29/70)	65.7% (46/70)	34.3% (24/70)
NSG	96% (48/50)	4% (2/50)	94% (47/50)	6% (3/50)

**Table 6.** Performance in non-d-linked subject–object *wh-*interrogatives (percentages, with *n* in parentheses).

Group	Subject		Object	
	Target	Non-target	Target	Non-target
IMG	68.2% (58/85)	31.8% (27/85)	78.9% (67/85)	21.1% (18/85)
AVG	82.8% (58/70)	17.2% (12/70)	87.2% (61/70)	12.8% (9/70)
NSG	96% (48/50)	4% (2/50)	98% (49/50)	2% (1/50)

As Table 5 shows, the IMG participants performed less significantly than the AVG participants in both subject and object d-linked *wh-*interrogatives [IMG: (subject) 42.4%; and (object) 54.1%; AVG: (subject) 58.6%, and (object) 65.7%, accuracy]. It is also clear that both learner groups performed better in object d-linked *wh-*interrogatives than in subject d-linked ones. However, both groups differ significantly from the control group.

Table 6 shows that both learner groups do not differ significantly; they both fare somehow better than in d-linking condition, particularly in object contexts [IMG: (subject) 68.2%, and (object) 78.9%; AVG: (subject) 82.8%, and (object) 87.2%, accuracy].

The overall results in both d-linking and non-d-linking conditions, presented in Tables 5 and 6, show that the control group performed as expected. They rejected the resumptive pronouns in ungrammatical sentences and accepted gaps in grammatical ones. Both learner groups fare in non-d-linked better than in d-linked *wh-*interrogatives. This shows that non-d-linked *wh-*interrogatives are easier to acquire than d-linked ones, which in turn implies that they have encountered less difficulty in the syntax proper data than that at the syntax-discourse interface.

As for the d-linking condition, the two experimental groups performed significantly less than the control group (cf. Table 5). A One-way ANOVA performed on the d-linked *wh-*interrogatives revealed statistically significant differences between the control group and each of the learner groups on both subject and object conditions *F*(2, 38) = 91.248, *p* <.001], and *F*(2, 38) = 19.961, *p* < .001], respectively.

For comparisons within groups, Post hoc Scheffe test indicated high statistically significant differences between the control group and each of the learner groups [IMG: (subject) *MD* = 4.364, *p* <.001, *p* <.05; (object) *MD* = 5.723, *p* <.001, ( *p*<.05); AVG: (subject) *MD* = 2.417, *p* =.01, ( *p* <.05), (object) *MD* =3.351, *p* =.01)]. ^12^

As for the learner groups, there were significant differences between the intermediate and advanced learners in both subject and object conditions [IMG: (subject), *MD* =3.907, *p* <.001, ( *p<.*05), (object) *MD* = 3.037, *p* <.001), ( *p<.*05); AVG: (subject) *MD* =3.015, *p* <.001), ( *p* <.05), (object) *MD* = 1.907, *p* = .01, ( *p* <.05)].

The above findings in terms of the d-linking condition clearly indicate that both learner groups performed far short of the control group. Put differently, both learner groups seem to accept resumptive pronouns in d-linking condition in ungrammatical *wh-*interrogatives, and reject gaps in grammatical ones, which results in bad performance. They also show clearly that the advanced group performed better in the object condition than the intermediate group.

Turning now to the non-d-linking condition, the intermediate learners and the advanced learners fare better in the object condition. However, they both differ significantly from the control group, though they performed better than they did in the d-linked *wh-*interrogatives (cf. Table 6). A one-way ANOVA conducted on the non-d-linked *wh-*interrogatives indicated that there were statistically significant differences between the control group and the learner groups each [ *F*(2, 38) = 88.985, *p* <.001] for subject, and [ *F*(2, 38) = 4.119, *p* <.024] for object.

As for comparison within groups, Post hoc Scheffe test revealed statistically significant differences between the control group and the intermediate group [IMG: (subject) *MD*= 9.623, *p* <.001, ( *p<.*05); (object) *MD* = 1.764, *p* =.01, ( *p* < .05)]. However, there were no significant differences between the control group and advanced group [AVG: (subject) *MD*= .481, *p* = 1, ( *p* >.05), (object) *MD* = .341, *p* = 1, ( *p* >.05)].

This is an important finding of this study, because it highlights the better performance of the advanced group at the syntax proper. That is to say, they performed near-native like in judging the non-d-linked *wh*-interrogatives.

However, Scheffe test also revealed that there were no statistically significant differences between the intermediate group and advanced group [IMG: (subject) *MD*=1.714, *p* =.058, ( *p* >.05), (object) *MD* =.514, *p* =1, ( *p* >.05); AVG: (subject) *MD*=.462, *p* =1, ( *p*>.05), (object) *MD* = .317, *p* = 1, ( *p* >.05)].

This is also a significant finding due to the fact that both groups, intermediate and advanced learners, seem to perform near-native like in *wh*-interrogatives. This implies that the linguistic phenomena related to the syntax proper is easier to acquire.

The overall findings reached regarding the d-linking condition suggest that: i) both learner groups, though with a varying degree in favor of the advanced group, performed better in the non-d-linked than they did in the d-linked *wh-*interrogatives, indicating that (a) they have acquired the syntax proper linguistic phenomena, and (b) the fact that the advanced learners performed like native speakers indicates that they face no difficulty in processing the non-d-linked *wh*-structures, ii) both learner groups, particularly the advanced learners, performed much better in both ungrammatical and grammatical *wh*-questions, which suggests that they rejected resumptive pronouns and accepted gaps, respectively, relatively close to native speakers’ performance in the non-d-linking condition, and iii) the fact that the advanced group performed like native speakers in the non-d-linked, but far less than them in the d-linked *wh-*questions shows clearly that they have acquired the proper way of using the [-DL], but not [+DL] features, and iv) the fact that both groups performed far better in the non-d-linked than in the d-linked *wh-*questions indicates that they encountered considerable difficulty in processing or analyzing linguistic phenomena in the d-linking condition, or more specifically, at the syntax-discourse interface.

## 6. Discussion

The overall results reached revealed that the intermediate and advanced learners performed significantly differently from the native speakers in the d-linking condition, and significantly far less than they did in the non-d-linking condition, in both subject and object contexts. This indicates that both groups accepted the resumptive pronouns in the ungrammatical, but did not accept gaps in the grammatical *wh-*interrogatives. This otherwise clearly indicates that they were successful in acquiring the syntax proper *wh-*questions, but were not so in acquiring the syntax-discourse ones, thus supporting the IH. Thus, I will discuss the results of the study in terms of:

i. learners’ performance in d-linking *versus* non-d-linking conditions,
ii. learners’ performance in subject *versus* object contexts and,
iii. Access to UG and L1 interference

As for (i), given the statistics in section 5 above, briefly repeated below for convenience, both learners performed in the d-linking condition statistically significantly less than the control group [IMG: (subject) 42.4%; and (object) 54.1%; AVG: (subject) 58.6%, and (object) 65.7%, accuracy] (cf. Table 5). It is also clear that advanced learners performed better than intermediate learners. A One-way ANOVA performed on the d-linked *wh-*interrogatives revealed statistically significant differences between the control group and each of the learner groups both in subject and object conditions [ *F*(2, 38) = 91.248, *p* <.001], and *F*(2, 38) = 19.961, *p* < .001], respectively.

As for the non-d-linking condition, both learner groups performed much better than they did in the d-linked *wh-*questions [IMG: (subject) 68.2%, and (object) 78.9%; AVG: (subject) 82.8%, and (object) 87.2%, accuracy] (cf. Table 6). This means that both learner groups have acquired the syntax proper in which these non-d-linked *wh-*interrogatives are processed and analyzed, more specifically in object contexts. A one-way ANOVA conducted on the non-d-linked *wh-*interrogatives indicated that there were statistically significant differences between the control group and the learner groups each [ *F*(2, 38) = 88.985, *p* <.001] for subject, and [ *F*(2, 38) = 4.119, *p* <.024] for object.

Post hoc Scheffe test, however, shows that there were no significant differences between the control group and advanced group [AVG: (subject) *MD*= .481, *p* = 1, ( *p* >.05), (object) *MD* = .341, *p*= 1, ( *p* >.05)]. As noted so far, this is a very significant result which clearly highlights the fact that both learner groups performed near-native like in non-d-linked constructions, which in turn indicates that they have acquired the syntax proper relatively well.

Regarding (ii), the fact that both learner groups’ performance in object condition is far better than in subject condition is not surprising. Given the syntactic facts of YA: i) YA is a null subject language, ii) YA does not use *wh-*movement strategy, but rather base-generation one, and iii) YA does not exhibit *that-t* effects, this finding is again not surprising. Given the base-generation strategy utilized by YA, and the assumption that resumptives are the spell out of (uninterpretable) agreement features and Case (cf. Alexiadou and Anagnostopoulou 1998), resumptive pronouns are likely to persist in L1 YA learners’ endsate grammars, or their Interlanguage (cf. Lardiere 2009). Thus, when the *wh-*word/phrase is base-generated in the topmost Spec, CP, these agreement features and Case are likely to spell out as resumptives, which is not the case in English, which in turn indicates that learners will have a “tendency” to accept these resumptives in their English production.

The fact that YA does not exhibit the *that-t* effects results in YA learners being unsensitive to such effects in English. If this is on the right track, the learners will accept resumptives in ungrammatical *wh-*questions, and will not accept gaps in grammatical ones. Given that *that-t* effects are related to subject *wh-*questions, but not to object ones, their bad performance in subject *wh-*questions could be a direct result of that.

Another possible interpretation of this subject-object asymmetry is to look at the nature of the object *wh-*questions in English, *i.e.* English *wh-*questions allow the complex ‘that-he/it’, which is not different from YA. Examples in (3a) and (3b) illustrate the point in question.

(3) a. ʔayyan dars qaal ʕali *(ʔinn-uh) katab*(-uh)?      D-linked

    which lesson said  Ali that-he    wrote-it

    ‘Which lesson did Ali say that he wrote (*it)?

  b. mu qaal ʕali *(ʔinn-uh) ?aštri*(-uh) ?           Non-D-linked

    what said Ali that-he bought-it

    ‘What did Ali say (that) he bought (*it)?

Thus, the only difference between YA and English in object - *wh-*questions turns to be the obligatory-optional use of the resumptive pronoun *it*, which suggests that YA learners find object *wh-*questions easier to process/acquire than subject ones. This may be thought of as a “facilitator” aiding the learners to perform better in object *wh-*questions than in subject ones. In fact, the subject-object asymmetry found in our study provides support to previous studies (see *e.g.* Tsimpli and Dimitrakopoulou 2007, and the references there).

As for (iii), considering the nature of the data utilized, they are complex *wh-*interrogatives, involving long-distance *wh-*movements, because they each consist of a matrix and complement clause. These complex *wh-*interrogatives involve resumptive pronouns “as a saving device to redeem derivations and structures that would otherwise violate a fundamental principle of the grammar” ( Rouveret 2011: 2, see also Leal Méndez and Slabakova 2012). These structures are expected to be more difficult to process or analyze by the learners, specifically if the L1 does not use *wh-*movement strategy and allows resumptive pronouns. Put differently, the *wh-*movement in these complex structures will undergo several cyclic *wh-*movements from the base position to the topmost Spec, CP. Thus, learners will face considerable difficulty in processing these A’-chain *wh-*questions much more than that faced in processing simple *wh-*questions (i.e. one-clause *wh-*questions). ^13^

At the level of the syntax proper, *i.e.* non-d-linked *wh*-questions, L2 learners in this study performed approximately well. The fact that they performed well, at least at the syntax proper indicates that the learners, specifically the advanced learners, have acquired how resumptive pronouns are used in English (non-d-linked) *wh-*interrogatives, by rejecting these pronouns in ungrammatical sentences and accepting gaps/empty categories in grammatical ones. This is evidence that the UG activated by the learners’ L1 (before puberty, cf. Pinker 1995; Shormani 2014c) has worked its path in L2 acquisition, and is still accessible, allowing them to re-set the resumptive parameter according to L2 grammar in the syntax proper. Form a minimalist perspective, L2 learners were able to use *Merge* and *Agree* operations properly. As for *Merge*, one could argue that learners, specifically the advanced ones, were able to apply the internal *Merge* ( *wh*-movement) accurately, and the *wh-*movement was applied cyclically, involving several *wh-*movement stances. Assuming with Chomsky (2000, 2001), for instance, that the head C has an EPP (Extended Projection Principle) (or [ *u*wh]) feature, EPP triggers the *wh*-word/phrase to remerge in Spec, CP, and the learners seem to have acquired it. Regarding *Agree,* it is possible to argue that L2 learners have acquired where to or not to apply it in YA and English *wh-*questions. That is to say, while *Agree* applies in YA resulting in resumption, learners did not apply it in English (non-d-linked) *wh-*questions.

However, at the syntax-discourse interface, L2 learners involved in this study fail to apply the parameter re-setting, or *Merge* and *Agree* operations successfully. In other words, L2 learners were not able to re-set the parameters of L1 according to L2, in such a way as to reject resumptive pronouns in their L2 grammars at the syntax-discourse interface, i.e. when discourse comes to play. The fact that [+DL] features are imposed by discourse such as discourse (co)referentiality (see *e.g.* Shormani 2017), context, situation, etc. in resumption, for instance, to disambiguate a grammatical structure, or what has been referred to by Tsimpli and Dimitrakopoulou 2007) as “garden-path effects”, manifests itself clearly in their L2 vulnerability. This suggests that L2 learners’ grammar cannot reach endstate or native speakers’ competence, which also suggests that access to UG is partially restricted, the reason of which might be attributed to L1 interference, among other reasons. ^14^ One could assume that though these L2 learners have reached high level of learnability in I-language of L2 (cf. Chomsky 2013), they were just unable to re-set the parameters concerning the use of resumptive pronouns in L2 English. From a minimalist perspective, it seems that L2 learners even at advanced levels are not able to distinguish where to apply the operations *Merge* and *Agree* appropriately in their performance. Put simply, they were unable to figure out that a resumptive pronoun cannot be merged with a moved *wh-*word/phrase in L2 English, and while *Agree* is spelled out as a resumptive pronoun in YA (cf. Alexiadou and Anagnostopoulou 1998, see also Tsimpli and Dimitrakopoulou 2007, for Greek), it is not the case in English *wh*-questions at the syntax-discourse interface. Having this deficit characterization of *wh-*interrogatives at the syntax-discourse interface, L2 learners fail to perform like native speakers, as predicted by the IH.

This makes it clear, though, that L1 interference may play a role in the learners’ performance. That is, the difficulty encountered by L2 learners could be attributed to L1 interference in acquiring linguistic phenomena relating to the syntax-discourse interface. Or, L2 learners find it difficult to process and analyze a particular structure involving discourse-domain related phenomena, thus, resulting in resorting to their L1, as a “subconscious” strategy to overcome such difficulties ( Shormani 2014a & b). The resumption strategy of YA could be said to have been transferred to L2 counterpart structures, hence resulting in ungrammatical performance. Assuming with Alexiadou and Anagnostopoulou (1998) and Tsimpli and Dimitrakopoulou (2007) that features like subject-verb agreement and Case are uninterpretable features and spell out as resumptive pronouns, it could be argued that these features are transferred from L1 YA to L2 English at the syntax-discourse interface. This L1 interference could also be said to make L1-L2 counterpart *wh*-structures “difficult to identify and analyse in the L2 input due to persistent, maturationally-based, L1 effects on adult L2 grammars” ( Tsimpli and Dimitrakopoulou 2007: 217).

## 7. Conclusions and further implications

This study examined L2 acquisition at the syntax-discourse interface by L1 YA learners acquiring English *wh*-interrogatives, at both d-linking and non-d-linking conditions, or syntax proper and syntax-discourse interface, respectively. The aim was to answer one major question: do linguistic phenomena relating to syntax-discourse interface constitute difficulty for Yemeni learners of English? According to the results reached, it is clear that the answer is *yes;* L1 YA learners’ competence of English at the syntax-discourse interface is still vulnerable.

In our corpus, I found strong support to IH predictions, *i.e.* L2 acquisition is vulnerable at the syntax-discourse interface. Both groups of learners, intermediate and advanced, involved in this study found considerable difficulty in acquiring the *wh-*interrogatives at the syntax-discourse interface. However, they performed significantly better in the syntax proper, *i.e.* the non-d-linked *wh-*questions. Given this, our study, thus, provides strong support to previous studies on this phenomenon (see *e.g.* Tsimpli and Sorace 2006; Sorace and Filiaci 2006; Tsimpli and Dimitrakopoulou 2007; Tsimpli and Mastropavlou 2007; Sorace and Serratrice 2009; Sorace 2005, 2006, 2016).

In addition, our study provides important findings that could be employed by university teachers while prescribing textbooks for their students, or even while their teaching of syntax courses. In the latter aspect, university teachers are advised to involve comprehensive comparisons of YA with English *wh*-question formation in both languages, concerning specifically the different *wh*-mechanism and resumption strategies utilized by each language. In this way, teachers could provide students with linguistic input sufficient for acquiring such differences, and students would be made aware of these differences between their L1 and L2 they are acquiring from early stages of their acquisition, and more specifically before these structures get fossilized.

To conclude, it is true that the results of this study support numerous previous studies, but these studies were conducted in different contexts, different L1s, and L2s, different from those of this study. Thus, it goes without saying that further research be conducted to see whether the results concluded with in this study are supported, not only concerning *wh*-interrogative acquisition, but also including acquisition of other linguistic phenomena such as articles, focus, topicalization, subject-verb agreement, etc. in English, and I leave these for future research.

## Data Availability

*Figshare*: L2 Acquisition of Wh-interrogatives at the Syntax-discourse Interface: Interface Hypothesis again. *F1000Reseacrh.* https://doi.org/10.6084/m9.figshare.23792385.v5. (Shormani, Mohammed. 2023) This project contains the following underlying data:
• Underlying data-total results.pdf. (The tabulated results indicating the group (IMG, AVG & NSG), item number, number of responses and D-linking condition: d-linked and non-d-linked *wh*-interrogatives). Data is available under the terms of the terms of the Creative Commons Attribution 4.0 International license (CC-BY 4.0). *Figshare*: L2 Acquisition of Wh-interrogatives at the Syntax-discourse Interface: Interface Hypothesis again. *F1000Reseacrh.* http://dx.doi.org/10.6084/m9.figshare.23792385. (Shormani, Mohammed. 2023) This project contains the following extended data:
• Sentence.order: • Example of participants’ responses (Intermediate Learner Group (IMG)) • Example of participants’ responses (Advanced Learner Group (AVG)) • Example of participants’ responses (Native Speaker Group (NSG)) Any further individual responses can be requested from the author. Data utilized in this article are available under the terms of the Creative Commons Attribution 4.0 International license (CC-BY 4.0).

## Footnotes

^1^
Null/ *pro* subject is a covert subject, i.e. not phonetically realized as in the Arabic sentence in (i): (i) kataba d-darsa wrote the-lesson ‘He wrote the lesson.’ In (i), the subject in Arabic is considered to be *null/pro*, because Arabic allows the subject pronoun to be dropped.

^2^
Base-generation strategy is a syntactic process in which the *wh*-word does not move to its final position, viz., Spec,CP, but rather first merges in this position and remains there throughout the derivation.

^3^
In early 1980s, Chomsky proposed the Principles and Parameters Framework as a theoretical solution to several problems including what knowledge of language is, and how humans acquire it. He proposed that humans are innately, genetically and tacitly endowed with a Language Faculty, which contains UG ( Chomsky 1981). UG, Chomsky argues, contains two types of linguistic/syntactic rules, *viz.*, Principles and Parameters. Principles are rules universal to all human languages *e.g.* Extended Projection Principle, which states that every clause must have a subject. Parameters, on the other hand, are syntactic rules specific to every language, *e.g.* Null Subject Parameter, which states that, in some languages, the subject could be null/covert, while in others it is overt (see *e.g.* Shormani 2014b & c, 2016).

^4^
As will be discussed in subsequent sections of this article, this study examines this view as well. It argues that this Partial Access to UG is due to L1 interference/persistence in L2 endstate grammar. In other words, L1 influence restricts the access to UG only in relation to linguistic phenomena relating to syntax and external interfaces such as syntax-discourse/pragmatics.

^5^
With the view that in minimalism language acquisition is acquisition of features, there have been several calls that SLA studies, particularly those done within the generative approach, should be conducted at interfaces (see *e.g.* Marantz 1995; Chomsky 2005; Lardiere 2009). This is due to the fact that most of the difficulties encountered by L2 learners have been attributed to these interfaces, be they within syntax and its interfaces or within other modules and their interfaces.

^6^
This line of thought is also supported by Reinhart (2006). She proposes that the interface difficulties encountered by L2 learners are caused by reference set computations, resulting in non-native like performance/proficiency.

^7^
However, a recent study conducted by Jin *et al.* (2022) claims exactly the opposite. Their study’s purpose was to examine the adult heritage speakers’ mastery of referential nominal expressions regulated at the syntax-semantics and syntax-discourse interfaces in Mandarin Chinese. The participants of the study are 58 adult heritage speakers of Chinese from overseas and 29 native speakers of Chinese. In their study, they found that their heritage participants performed in linguistic phenomena relating to syntax- discourse interface better than they did in linguistic phenomena relating to syntax- semantics interface.

^8^
The assumption that *wh- *phrases are considered d-linking constituents is advocated in several studies ( *e.g.* Pesetsky 1987, 2000; Aoun & Li 2003). The term *d-linking* was first proposed by Pesetsky (1987) holding that it is a discourse property. Pesetsky (1987, 2000) argues that a *wh- *phrase is considered a D-linked constituent if the question introduced by it requires an answer which individualizes an entity or a set of entities from that previously referred to in the discourse, understood to both interlocutors of, say, a conversation. Thus, it should be noted that English and YA use similar *wh- *phrases for d-linking and non-d-linking: YA uses the *wh- *phrase *ʔayyan-NP* (English *Which-NP *) for subject and object d-linking, and *manu* and *mu* (English *Who* and *What,* respectively) for non-d-linking, subject and object, respectively.

^9^
See also Tsimpli and Dimitrakopoulou (2007), for somewhat similar data from Greek, however the differences between Greek and YA. Greek, for example, uses *wh*-movement strategy, and allows (in)animacy effects while YA use base-generation strategy and does not allow (in)animacy, among other differences.

^10^
Note that the *wh*-data were presented to the participants randomly, i.e. without numbering. The numbers in Table 2 indicate the order in which the *wh*-word/phrase occurs in the questionnaires.

^11^
This is the link to the OUMG Test: https://www.press.umich.edu/elt/compsite/cleargrammar/CG_part1

^12^

*MD* = Mean Difference (I-J)

^13^

One-clause *wh*-questions in YA can be exemplified in (i), in which we observe that the complex ‘that-he/it’ is obligatorily absent in YA examples, exactly like English ones. Thus, it is obviously clear that such English one-clause *wh*-questions could be easier for L2 learners to process and analyze than those consisting of matrix and complement clauses, like the ones utilized in this study. (i) ?ayyan  rajjaal (*ʔinn-uh)  jaaʔ ?ams ?  which   man (*that-he)  came yesterday  ‘Which man (*that he) came yesterday?’

^14^
Somewhere else, I argued that UG is available and accessible to language learners alongside their lifespan, provided that it has been activated by L1 before puberty (see *e.g.* Pinker 1995). The learners’ inability to re-set the UG parameters, or apply *Merge*, *Agree* (or even *Move*, Internal Merge) operations according to L2 ones cannot be ascribed to accessibility to UG *per se.* However, this inability could be ascribed to non-linguistic factors such as residual optionality caused by, for instance, insufficient linguistic input, the way this linguistic input was presented to the learners in (or out of ) classroom, and/or the deficit competence of those who present it, among many other reasons irrelevant to UG such as “age, motivation, attitudes towards L2 and its speakers, interest, etc.” ( Shormani 2014c: 84).
