# Supplementary material for: L2 acquisition of Wh-interrogatives at the syntax-discourse interface: interface hypothesis again
Source: F1000Res. 2023 Sep 25;12:1198. [Version 1] doi: 10.12688/f1000research.133802.1 (PMC10618936; doi:10.12688/f1000research.133802.1)
Supplement: Peer review history

# Reviewer report: Reviewer response for version 1

**DOI:** 10.5256/f1000research.146815.r210086

**Published:** 2023-10-30

**Authors:** Wagdi Rashad Ali Bin-Hady

**Affiliations:** 1 Department of English, Hadhramout University, Mukalla, Yemen

This research work is original. The researcher examined the L2 acquisition of *Wh-*interrogatives in three learner groups. I strongly recommend the work for indexing after mangling a few comments:

1. The first sentences in the introduction need justification, so you are recommended to add some sources.
2. The author is required to mention some of the previous studies which have been conducted on YA to set this research in its field. 
3. Some paragraphs should be shifted from the introduction into other sections e.g.,
*"It involves three groups of participants (n=41): intermediate learner group (IMG, n=17), advanced learner group (AVG, n=14) and control group (NSG, n=10). The intermediate learners are BA English students, at their fourth year, and the advanced learners are MA English students (after completing three semesters of courses, and before commencing writing their MA Theses). Both learner groups are native speakers of YA, and the criterion followed to determine their proficiency in English is the Online University of Michigan grammar (placement) Test (OUMG) parts 1-4. The control group are native speakers of (British) English, MA students at the University of Essex."*
4. Under the method section, add research design. 

Is the work clearly and accurately presented and does it cite the current literature?

Yes

If applicable, is the statistical analysis and its interpretation appropriate?

Yes

Are all the source data underlying the results available to ensure full reproducibility?

Yes

Is the study design appropriate and is the work technically sound?

Partly

Are the conclusions drawn adequately supported by the results?

Yes

Are sufficient details of methods and analysis provided to allow replication by others?

Yes

Reviewer Expertise:

Applied lingustics

I confirm that I have read this submission and believe that I have an appropriate level of expertise to confirm that it is of an acceptable scientific standard.

# Reviewer report: Reviewer response for version 1

**DOI:** 10.5256/f1000research.146815.r210085

**Published:** 2023-10-19

**Authors:** Arif Ahmed Al-Ahdal

**Affiliations:** 1 Department of English Language, College of Arts and Sciences, Qassim University, Methnab, Saudi Arabia

This paper addresses L2 acquisition, focusing on acquiring English wh-interrogatives by L1 Yemeni learners at the syntax-discourse interface; it examines interface hypothesis proposed by Sorace and colleagues (2006). The study findings indicate that the learners’ endstate knowledge of wh-interrogatives is vulnerable at the syntax-discourse interface, while their knowledge at the syntax proper is not. These findings support a wide range of findings reached and articulated by a great number of studies in this area of language study in general, and L2 acquisition, in particular. Considering the Yemeni context, the study contributes greatly and substantially to the body of research on L2 acquisition process, in general, and acquisition of wh-interrogatives, per se.

 The paper develops the subject matter in an academic and logical way, which vividly mirrors the author’s sound knowledge in this area of research, and syntax interfaces with other cognitive modules of the grammar. It is well-written, with no grammar or stylistic problems.

 The originality of topic researched is obvious as the topic is not over-researched,  and the findings arrived at would be of great significant to all those interested in the field. A valuable and current bulk of related literature has been consulted and cited, comparing this study to other studies in terms of context, participants, instrument, approach, method of analysis and findings. The introduction section provides a comprehensive account of the nature of the study, scope, purpose, research design, and the gap it tends to fill. The conclusion section skillfully summarizes the whole paper and its findings, and provides tertiary-level instructors  with salient pedagogical implications that could be applied in teaching syntax course(s) in order  to improve the acquisition process of syntactic phenomena, specifically wh-interrogatives.

 There are, however, three minor comments to be considered and addressed before the manuscript is indexed. These are:

1. The following example, given in Footnote 13, should be incorporated into the text:
  (i) ?ayyan  rajjaal (*ʔinn-uh) jaaʔ  ?ams ?
       which    man (*that-he)   came  yesterday
      ‘Which man (*that he) came yesterday?’
2. Inconsistency:
  i) Phonetic symbols: the question mark /?/ is sometimes used instead of the phonetic symbol /ʔ/, which should be used throughout the Arabic data.
  ii) Comma “ , ” in in-text referencing: there are few instances where “ , ” between the author’s name and date as in “(…Sorace *et al*., 2009)” is used. This should be fixed. There are also a few instances of using “&” instead of “and” between two authors’ names as in “…Aoun & Li 2003”, in Footnote 8, which should be checked throughout.
3. Fragment: there is a fragment in the introduction section: “And third, the paucity of studies in Yemeni context that tackle this important aspect of L2 acquisition research”, The verb “ *is” is missing and “the” should be added before “Yemeni*. It should, thus, be rewritten as “And third is the paucity of studies in the Yemeni context that tackle this important aspect of L2 acquisition research.”

**Apart from the flaws referred to above, I find that the work is in a good shape and would certainly be a valuable reference for English syntacticians and syntax practitioners across the globe.**

Is the work clearly and accurately presented and does it cite the current literature?

Yes

If applicable, is the statistical analysis and its interpretation appropriate?

Yes

Are all the source data underlying the results available to ensure full reproducibility?

Yes

Is the study design appropriate and is the work technically sound?

Yes

Are the conclusions drawn adequately supported by the results?

Yes

Are sufficient details of methods and analysis provided to allow replication by others?

Yes

Reviewer Expertise:

Professor of Applied Linguistics

I confirm that I have read this submission and believe that I have an appropriate level of expertise to confirm that it is of an acceptable scientific standard.
